# Special Issue “The Role of Carotenoids in Health and Disease”

**DOI:** 10.3390/ijms262211050

**Published:** 2025-11-15

**Authors:** Nikolay E. Polyakov, Lowell D. Kispert

**Affiliations:** 1 Institute of Chemical Kinetics and Combustion, 630090 Novosibirsk, Russia; 2 Department of Chemistry, University of Alabama, Tuscaloosa, AL 35401, USA

Carotenoids represent a ubiquitous and critically important class of natural isoprenoid pigments, synthesized de novo by plants, algae, and photosynthetic bacteria [1,2,3]. Their vibrant red, orange, and yellow hues are not merely for aesthetic purposes; they play fundamental roles in photoprotection and light harvesting within photosynthetic organisms. In the realm of human health, the biological significance of carotenoids, which must be acquired through the diet, has expanded far beyond their initial recognition. This Special Issue, entitled “The Role of Carotenoids in Health and Disease,” features a collection of articles that describe cutting-edge research and emerging developments in this field. Topics covered include the features of biosynthesis and degradation of carotenoids; the health benefits and antioxidant functions of carotenoids; their application in cancer, and cardiovascular diseases; interaction of carotenoids with lipid membranes; and the use of various delivery systems to enhance the stability and bioavailability of carotenoids. 

While the primary role of carotenoids is its potent antioxidant activity, encompassing the ability to quench singlet oxygen and scavenge peroxyl radicals, this is only one facet of a complex physiological profile. Individual carotenoids, such as β-carotene, α-carotene, and β-cryptoxanthin, function as crucial provitamin A precursors, which are metabolized into retinal and retinoic acid, compounds essential for vision, cell differentiation, and immune function. Beyond this, specific carotenoids like lutein and zeaxanthin accumulate in the macula lutea, protecting the retina from phototoxic blue light, while others demonstrate functions in cell membrane stabilization by influencing lipid packing and fluidity and in direct immune modulation by influencing lymphocyte proliferation and cytokine signaling. This multifaceted bioactivity has led to intensive research into their therapeutic potential, particularly concerning their interactions with free radicals. A growing body of evidence underscores their potential to mitigate the pathogenesis of major diseases, including myocardial infarction, cerebral thrombosis, and various neoplasms [3,4,5]. The common thread linking these conditions is the pervasive damage inflicted by reactive oxygen species (ROS) and oxidative stress on cellular lipids, proteins, and DNA. Carotenoids, acting as a first line of defense, offer a compelling nutritional strategy to modulate these pathways and potentially slow disease progression.

However, a profound paradox exists between the demonstrated therapeutic potential of carotenoids and their viable pharmaceutical application. This “carotenoid conundrum” is rooted in a suite of challenging inherent physicochemical properties:

- Extreme lipophilicity: Their highly hydrophobic, long conjugated polyene chain renders them virtually insoluble in aqueous biological fluids, leading to poor and variable oral bioavailability. This property also complicates parenteral administration, as they cannot be formulated in conventional intravenous solutions without risking precipitation or embolism.
- Profound chemical instability: The very feature that confers their antioxidant capacity, namely the conjugated electron system, makes them exceptionally susceptible to degradation. In the presence of oxygen, light, heat, and acids, they readily undergo oxidation and isomerization, losing their bioactivity and color.
- Pronounced photosensitivity: Exposure to light can rapidly degrade carotenoids, making storage, handling, and formulation into stable dosage forms a significant technical hurdle.

These challenges directly impede the effective use of carotenoids not only as therapeutic antioxidants but even as natural colorants in foods and supplements. The development of advanced, robust delivery systems is a critical and non-negotiable prerequisite for translating the preclinical promise of these low-molecular-weight, highly lipophilic compounds into clinical reality [6,7].

All articles included in this Special Issue, which are briefly summarized below, can be found via the following link: https://www.mdpi.com/journal/ijms/special_issues/YKFB4DDCZ1, accessed on 14 November 2025. Four review articles and four research articles are presented in this Special Issue.

The first review entitled “The endless world of carotenoids—structural, chemical and biological aspects of some rare carotenoids” focuses on various physicochemical properties of carotenoids, including its ability to form supramolecular “host–guest” complexes with water-soluble drug delivery systems, and their participation in various model redox processes [Contribution 1]. The application of electron paramagnetic resonance (EPR) spectroscopy and density functional theory (DFT) calculations has elucidated the structural characteristics of carotenoid paramagnetic intermediates—including radical cations and neutral radicals—and their functional roles in natural systems. These studies provide critical insights into the potential biological activity of carotenoids and their health implications. Notably, some rare carotenoids, such as crocin, siphonaxanthin, and sioxanthin, exhibit distinct structural features compared to conventional carotenoids, including additional functional groups or unique group positioning outside cyclic structures (e.g., sapronaxanthin, myxol, deinoxanthin, and sarcinaxanthin). The incorporation of carotenoids into host matrices via strategic molecular design or self-assembly enables stabilization through multiple hydrogen and coordination bonds. This supramolecular integration results in improved stability, increased oxidation potentials, and enhanced antioxidant activity, coupled with tunable photo-oxidation kinetics. Furthermore, embedding carotenoids in nonpolar environments can significantly improve their photostability. Additionally, the use of nanoscale supramolecular delivery systems has shown promise in enhancing both the stability and bioactivity of both conventional and rare carotenoids, offering potential advancements in their therapeutic and nutraceutical applications.

Wenjing Su with co-authors presented a review titled “Improving the treatment effect of carotenoids on Alzheimer’s disease through various nano-delivery systems”, which comprehensively summarizes how nanotechnology can overcome the inherent limitations of carotenoids to enhance their therapeutic potential against Alzheimer’s disease [Contribution 2]. Various essential carotenoids, such as lutein, fucoxanthin, astaxanthin, lycopene and other, as well as rare carotenoids like crocin (natural pigments with potential antioxidant activity), demonstrate therapeutic potential in the management of various neurogenerative diseases, including Alzheimer’s disease (AD). However, their lipophilic nature and highly unsaturated structures contribute to poor aqueous solubility, low stability, and limited bioavailability, restricting their clinical application. To overcome these challenges, the development of nanoscale drug delivery systems has emerged as a promising strategy to enhance carotenoid efficacy. These systems can significantly improve solubility, chemical stability, membrane permeability, and bioavailability, thereby optimizing their therapeutic effects. Recent advances suggest that tailored carotenoid delivery platforms may offer neuroprotective benefits, potentially mitigating AD progression through enhanced bioactivity and targeted delivery.

M. Pasenkiewicz-Gierula and colleagues presented the review on computational studies examining the interaction of carotenoids with model lipid membranes. By leveraging molecular modeling techniques, such as molecular dynamics (MD) simulations, the work provides atomic-level insights into the dynamic and energetic principles governing how carotenoids incorporate into and influence bilayer structure and properties [Contribution 3]. The absorption and transport of carotenoids in the human body have been extensively investigated. This review begins by summarizing recent experimental advances in these processes, covering key aspects such as carotenoid carriers, systemic transport, tissue delivery, and molecular-level interactions with membrane receptors. The core focus of this review, however, lies in the computational exploration of carotenoid intercalation and dynamic behavior within lipid bilayers. These computational studies are particularly significant because they bridge microscopic molecular behavior with macroscopic ensemble properties, providing critical insights into the structure–function relationships of carotenoids. By employing molecular dynamics simulations and other computational approaches, researchers can elucidate the atomic-level interactions between carotenoids, lipid bilayers, and proteins. Such detailed analyses not only aid in interpreting the macroscopic physicochemical properties of carotenoids but also advance our understanding of their biological roles and functional mechanisms.

The next review presented by Tomas Bas explores the innovative applications of carotenoids and their transformative potential for human health and medicine [Contribution 4]. The work comprehensively examines the therapeutic promise of these compounds in managing chronic diseases, such as cancer, cardiovascular disorders, and age-related macular degeneration. The review places particular emphasis on novel protective mechanisms—extending beyond conventional antioxidant activity—and highlights the resulting innovative pharmacological benefits that underscore their growing clinical relevance. The author discusses emerging evidence on carotenoid-mediated pathways that modulate oxidative stress, inflammation, and cellular signaling, offering new avenues for disease intervention. A key focus of this work is the examination of cutting-edge advancements in carotenoid extraction and bioavailability enhancement. Innovations such as supramolecular encapsulation systems and nanotechnology-driven delivery platforms are shown to significantly improve the solubility, stability, and targeted absorption of these bioactive compounds. These technological breakthroughs not only ensure reproducible product quality but also enable the customization of carotenoid-based therapies to meet individual patient profiles, thereby advancing the paradigm of precision nutrition and personalized medicine. By synthesizing contemporary research with pioneering methodologies, this review provides a forward-looking perspective on the translational potential of carotenoids. It establishes a robust framework for future investigations, setting a new standard for interdisciplinary research at the intersection of nutritional science, pharmacology, and nanotechnology.

Galina Brychkova and colleagues studied the features of biosynthesis and degradation of carotenoids in lettuce (*Lactuca sativa* L.) from seedling to harvest. Lettuce (*Lactuca sativa* L.) is a globally significant leafy vegetable, yet its cultivars exhibit substantial variability in carotenoid concentrations at harvest [Contribution 5]. To elucidate the regulatory mechanisms underlying this variation, authors conducted integrated transcriptomic and metabolomic analyses on inner and outer leaves from six cultivars at distinct developmental stages. This approach identified critical gene-to-metabolite networks governing the accumulation of two nutritionally essential carotenoids: β-carotene and lutein. These findings reveal that differential expression of carotenoid biosynthetic enzymes drives cultivar-specific disparities in lutein and β-carotene production. Notably, sustaining high carotenoid levels in leaves requires precise regulation of metabolic sinks, including the diversion of β-carotene and lutein toward zeaxanthin and subsequently abscisic acid (ABA) biosynthesis. A key conclusion is that lettuce harvested at the commercially dominant maturity stage—often coinciding with onset of senescence—shows marked declines in carotenoids and other vital metabolites. Authors propose that earlier harvest of less mature plants could enhance the nutritional value of lettuce, optimizing its content of bioactive compounds for human health.

B. Tatarowska and colleagues investigated the influence of genetic and agronomic factors on the concentration of key antioxidant compounds, total carotenoids (TC) and vitamin C (VC), by a comprehensive analysis of 65 potato cultivars from 10 countries, (Contribution 6). Their findings demonstrated a highly significant effect of cultivar, harvest year, and tuber flesh color on TC content. Quantitatively, TC levels were markedly higher in yellow-fleshed cultivars compared to white-fleshed ones and exhibited a weak correlation with annual variations. A similar trend was observed for VC, which was also present at higher concentrations in yellow-fleshed potatoes. Furthermore, statistical analysis confirmed that the concentration of TC was a significant determinant of the total antioxidant activity in the potato tubers.

The modulation of carotenoid and phospholipid content in staphylococcus aureus membrane was studied by Laura Zamudio-Chávez with co-authors [Contribution 7]. The membranes of *Staphylococcus aureus* contain carotenoids produced during staphyloxanthin biosynthesis, which function as virulence factors by scavenging reactive oxygen species (ROS) and inhibiting antimicrobial peptides (AMPs). In this study, authors demonstrate that oxygen-restricted growth conditions in *S. aureus* downregulate carotenoid biosynthesis and alter phospholipid composition in both biofilms and planktonic cells, as revealed by LC-MS analysis. Under oxygen limitation, the biophysical properties of *S. aureus* membranes undergo significant changes, including the following:

- Increased lipid headgroup spacing;
- Reduced bilayer core order;
- Elevated liquid crystalline to gel phase transition temperature.

Notably, carotenoid-deficient membranes exhibit a highly ordered gel phase at low temperatures, indicating that carotenoids play a key role in maintaining membrane fluidity. These findings suggest that *S. aureus* in hypoxic environments (e.g., abscesses) likely contains reduced carotenoid levels, leading to modified membrane biophysical properties that may influence bacterial survival and pathogenicity.

The study by M. Rozanowska et al. characterized the efficacy of lutein, zeaxanthin, taurine, and melanin as scavengers of cation radicals generated from visual cycle retinoids [Contribution 8]. In the retina, retinoids critical for vision are continuously exposed to oxidative stress, and their oxidation products can have detrimental effects. Using pulse radiolysis, authors demonstrated that lutein and zeaxanthin efficiently scavenge retinoid radical cations with bimolecular rate constants approaching diffusion-controlled limits. While lutein demonstrates superior scavenging ability for retinoid radical cations in vitro, the physiologically high concentration of ascorbate in the retina positions it as the predominant protector of all visual cycle retinoids against oxidative degradation. Notably, α-tocopherol contributes substantially to the protection of retinaldehyde but exhibits significantly lower efficacy in safeguarding retinol and retinyl palmitate. Although the protective role of lutein and zeaxanthin appears limited in the retinal periphery, their antioxidant activity is substantially more pronounced in the macula, where their concentrations are highest.

The results of these studies on carotenoids indicate their great potential for treating diseases and promoting health. By forming supramolecular host–guest complexes, insoluble carotenoids are useful for water-soluble drug delivery systems. EPR measurements have detected the structure of paramagnetic forms that are important for understanding the mechanisms of carotenoids activity. The bioavailability of carotenoids in human body has been advanced by improved carotenoid carriers, systematic transport, tissue delivery, and membrane receptors. In conclusion, while the pathophysiological rationale for using carotenoids in modern medicine is strong, the bridge between their inherent biological activity and their effective clinical application must be built upon the foundation of sophisticated delivery platforms. The future of carotenoid therapeutics lies as much in the innovation of formulation science as it does in the continued elucidation of their biological mechanisms. For instance, through various nanoscale drug delivery systems, the treatment of Alzheimer’s diseases has been improved by carotenoids through carotenoid solubility, chemical stability, and membrane permeability. We expect that further discoveries in carotenoid research can eventually improve strategies for the treatment of a number of diseases.
